# The emerging roles of ribosome biogenesis in craniofacial development

**DOI:** 10.3389/fphys.2014.00026

**Published:** 2014-02-04

**Authors:** Adam P. Ross, Konstantinos S. Zarbalis

**Affiliations:** Department of Pathology and Laboratory Medicine, Institute for Pediatric Regenerative Medicine, Shriners Hospitals for Children, University of California at DavisSacramento, CA, USA

**Keywords:** ribosomopathies, neurocristopathies, neural crest, craniofacial development, ribosome biogenesis, TP53, cell cycle regulation

## Abstract

Neural crest cells (NCCs) are a transient, migratory cell population, which originates during neurulation at the neural folds and contributes to the majority of tissues, including the mesenchymal structures of the craniofacial skeleton. The deregulation of the complex developmental processes that guide migration, proliferation, and differentiation of NCCs may result in a wide range of pathological conditions grouped together as neurocristopathies. Recently, due to their multipotent properties neural crest stem cells have received considerable attention as a possible source for stem cell based regenerative therapies. This exciting prospect underlines the need to further explore the developmental programs that guide NCC differentiation. This review explores the particular importance of ribosome biogenesis defects in this context since a specific interface between ribosomopathies and neurocristopathies exists as evidenced by disorders such as Treacher-Collins-Franceschetti syndrome (TCS) and Diamond-Blackfan anemia (DBA).

## Introduction

Neural crest cells (NCCs) are a diverse population of migratory cells that give rise to most tissues during vertebrate development. Defects in NCC generation, migration, and differentiation are the underlying cause of a wide range of complex disorders known a neurocristopathies. In addition, NCCs to some extent, bear the major characteristics of stem cells including multipotency and self-renewal. Recent efforts have focused on the possibility of using neural crest-derived mesenchymal stem cells for regenerative purposes to address a plethora of degenerative disorders and injuries to tissues with limited capacity for repair and renewal. The regenerative use of these cells may become particularly important for disorders of the brain and skull. Consequently, a more thorough understanding of the extrinsic and intrinsic control mechanisms guiding NCC renewal and fate determination is needed before therapeutic approaches become applicable. Intriguingly, disorders caused by defects in ribosome biogenesis, also known as ribosomopathies, often affect specifically neural crest-derived tissues posing interesting questions as to the particular significance this basic cellular function holds for the NCC lineage.

## Clinical overview of neurocristopathies

**Figure 1:**
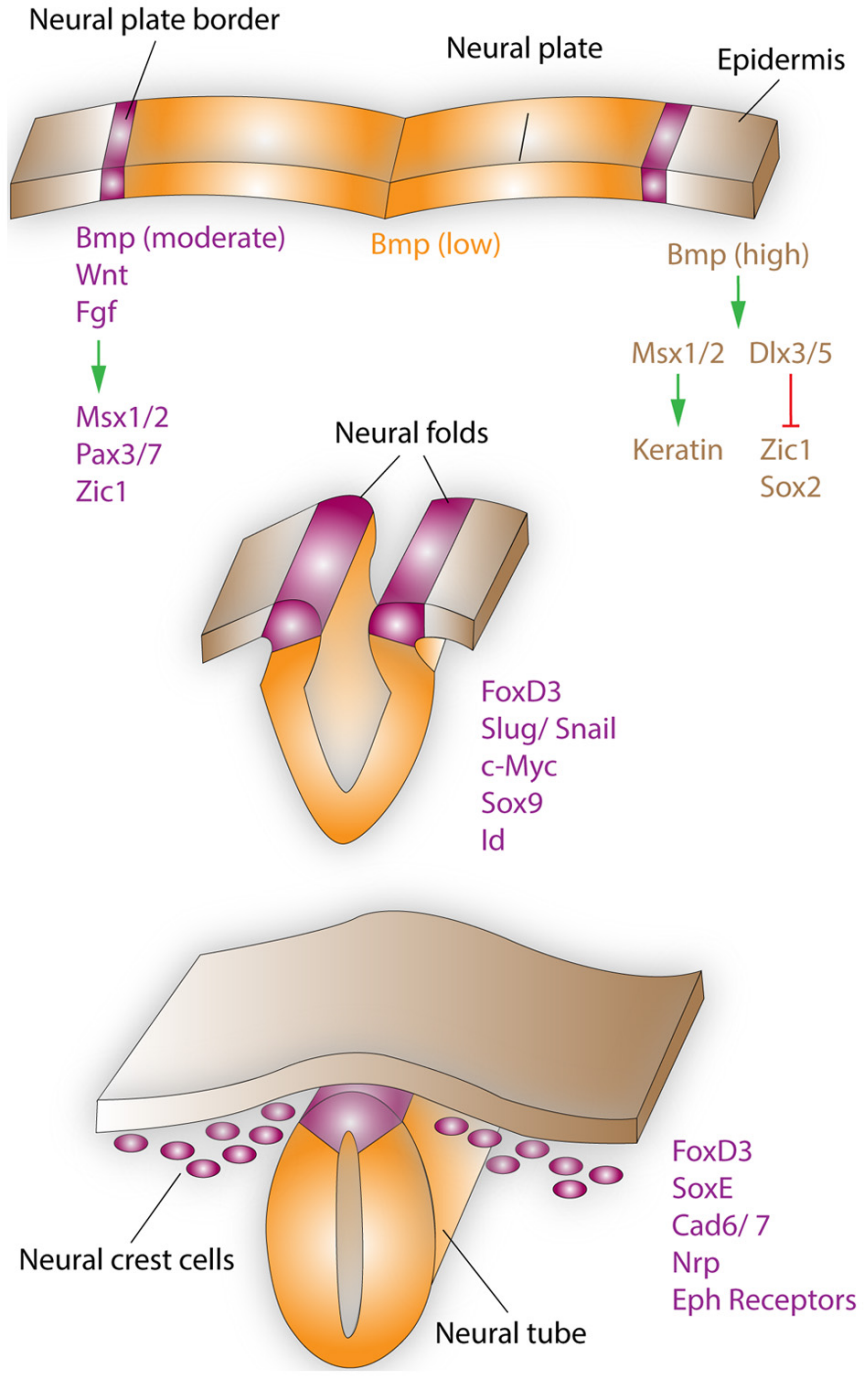
**Neural crest formation and migration during development**. Neural crest regionalization (top) at the boundary of the neural plate and epidermis is a multi-step process. First, the border of the neural plate is set via secretion of neural plate inductive signals (Fgf, Bmp, and Wnt) from the ventral ectoderm and paraxial mesoderm (not shown). Anteriorly, the timing of Bmp and Wnt signaling contributes toward setting the boundaries between epidermis, prospective neural crest, and neural plate. In the narrow band, where Wnt signaling induces Bmp signaling and Wnt signaling is not subsequently turned off, NCCs are formed. Bmp, Wnt, and Fgf, which are secreted by the prospective neural crest, induce the expression of border regionalization genes such as Msx1/2, Pax3/7, and Zic1. In contrast, in the epidermis high concentrations of Bmp induce the expression Msx1/2, which promote keratin expression and Dlx3/5, which induce Zic1 and Sox2 expression. Neural crest specification (middle) starts with the expression of FoxD3, Slug/Snail, c-Myc, Sox9, and Id by the border cells, which prevents this region from becoming either neural plate or epidermal tissue. EMT, delamination, and migration of NCCs (bottom), is primarily induced by FoxD3, Snail, and Sox9. These factors are also capable of inducing a cranial neural crest fate for cells of the lateral neural tube, when ectopically expressed in this region. After delamination NCCs migrate to their respective destinations, regulated by the expression of proteins such as FoxD3, SoxE, Cad6/7, Nrp, and Eph receptors. Specifically, the head and facial structures are largely products of the cranial neural crest, which is a mixed population of cells, with about 10% of these cells being multipotent progenitor cells.

During development of the human embryo, NCCs arise between the third and fifth weeks of gestation within the neural folds that separate the ectoderm from the neural plate (see Figure 1 for brief overview) (Etchevers et al., 2006). In the mouse, the mammalian model for neural crest development, the period of NCC generation ranges from embryonic day (E)7 to E11. The neural crest is a transient structure, with its cells undergoing an epithelial to mesenchymal transition (EMT), subsequent migration to a myriad of destinations, and eventual differentiation into many different cell types. The neural crest progenitor cell population gives rise to structures that are as diverse as neurons of the sensory and autonomic nervous systems, pigment containing cells of the epidermis, smooth muscle cells, as well as the bone, cartilage and connective tissue of the face and skull (Le Lievre and Le Douarin, 1975; Sauka-Spengler and Bronner-Fraser, 2008). The final fate of NCCs results from the interactions of a complex set of extrinsic signals with the internal environment of the cells, which can change during development in order to alter sensitivity to these extrinsic signaling factors (Gammill and Bronner-Fraser, 2002; Sauka-Spengler and Bronner-Fraser, 2008). For example, truncal NCCs, when transplanted at the vagal level will perform and differentiate into enteric neurons just as vagal NCCs, however they will release acetylcholine rather than catecholamines (Le Lievre and Le Douarin, 1975). Interestingly, NCCs that make up the structures of the head, face and jaw, appear to have some intrinsic properties, which are applied by the rostral endoderm, before they set out on their migration (Couly et al., 2002; Creuzet et al., 2002; Ruhin et al., 2003; Schneider and Helms, 2003). For example, patterning of the hyoid cartilage and the fate of cephalic NCCs depend on signals arising from the endoderm of the ventral foregut (Couly et al., 2002; Ruhin et al., 2003).

Neural crest disorders, known as neurocristopathies, are the result of aberrant proliferation, migration, differentiation, survival or any combination thereof of NCCs and lead to hypoplasia of organs and tissues precipitating a wide range of pathological conditions. The term “neurocristopathy” was first coined by Bolande in order to accentuate the commonalities of diverse afflictions (Bolande, 1997). Neurocristopathic hypoplastic malformations include many of the most common human birth defects such as Hirchsprung disease, cleft lip and/or palate, and conotruncal heart malformations. On occasion, these disorders can also show overlap with defects in neural tube closure. Isolated neural tube closure defects are not considered neurocristopathies, as closure events happen prior to the formation of NCCs. However, common mechanisms may underlie both neural tube closure defects and neurocristopathies.

Hirschsprung's disease, which is named after Harold Hirschsprung who reported the disorder in 1886, is a congenital malformation, which affects ~1 out of 5,000 children and is identified by the lack of enteric ganglia along a length of the intestine (Butler Tjaden and Trainor, 2013). The enteric ganglia, which are a component of the autonomic nervous system, include sensory neurons, motor neurons, and interneurons of the gut, which are critical for normal digestive processes and proper nutrition. Due to ensuing nutritional deficits of fat-soluble vitamins, this affliction was fatal until the 1940's, when a surgical method was first developed for restoring proper digestive function.

Waardenburg-Shah Syndrome is an exceedingly rare and genetically heterogeneous condition, occurring in 1 out of 500,000 live births. Affected individuals show both abnormal pigmentation and sensorineural deafness (Etchevers et al., 2006). The condition is caused by partial or complete loss of melanocytes in the skin and the stria vascularis, which is required for endolymph production and proper ion homeostasis in the scala media of the cochlea (Kruger et al., 2002).

Orofacial clefting is the result of improper outgrowth or fusion of the facial prominences and/or palatal shelves, which are derived from the maxilla of the first pharyngeal arch. Clinical examples of neurocristopathies that display facial clefting are Diamond-Blackfan anemia (DBA) and Treacher-Collins-Franceschetti Syndrome (TCS) (Trainor, 2010; Horos and von Lindern, 2012). TCS was first identified in 1900, and is characterized by hypoplastic development of the facial bones, especially the maxilla, mandible, and the zygomatic complex (Poswillo, 1975). In addition, TCS is often times associated with a cleft of the secondary palate. DBA is a rare, inherited bone marrow failure syndrome, characterized by normochromic macrocytic anemia, reticulocytopenia and deficiency or absence of erythroid precursors (Ito et al., 2010). Patients in roughly half of diagnosed cases of DBA also display craniofacial malformations (Horos and von Lindern, 2012). Intriguingly, the underlying molecular deregulation in both disorders are defects in ribosome biogenesis and the resultant inability of mutant cells to produce fully functional ribosomal particles that can properly participate in protein biosynthesis and cellular homeostasis.

Over the past 30 plus years however, the definition of neurocristopathies has changed to include not only pathologies of tissues directly derived from the neural crest, but also instances where altered NCC development hinders development of tightly associated tissues that are not themselves neural crest-derived, such as the heart or thyroid (Etchevers et al., 2006). Furthermore, syndromic neurocristopathies may display both tumorigenic and hypoplastic components. For instance, several types of tumors specifically associated with neurocristopathies, can occur with one another or with the underlying neurocristopathic affliction (Qualman et al., 1986; Jensen et al., 1993). One example of neurocristopathic tumors are neuroblastomas consisting of cells that resemble undifferentiated mesenchymal NCCs and acquire characteristics of neurofibromas or ganglioneuromas upon tumor regression. Originating from sympathetic components, neuroblastomas are the most common pediatric extracranial tumors, with very high rates of remission (~90%) (Nakagawara, 2004). Pheochromocytomas, derived from the chromaffin cells of the adrenal medulla, are a hallmark of a number of neural crest afflictions such as Hirschsprung's disease. Melanoma and Merkel cell carcinoma are cutaneous NCC cancers, which can be both invasive and aggressive in nature. Malignant melanoma arises in neural crest-derived melanocytes, which are distributed widely throughout the body as well as being one of the last tissues to differentiate, and can occur in any part of the skin, even without sunlight exposure. Pediatric melanoma is extremely aggressive and carries a high rate of metastasis. Merkel cells are found in the basal epidermis and together with sensory afferents form light-touch receptors. Merkel cells can give rise to carcinomas and have been shown to be neural crest derivatives even though a recent study suggests an epidermal origin (Szeder et al., 2003; Morrison et al., 2009). Merkel cell carcinomas are mostly localized in the dermis of the head and neck region, with limited spread over the body, and most commonly in the elderly. Merkel cell carcinomas are shown to associate with neurofibromatosis, breast and ovarian adenocarcinomas, as well as squamous cell carcinomas. Medullary thyroid cancers are also considered to be neurocristopathic tumors and can be diagnosed at all ages. In addition to the several types of isolated tumors associated with neurocristopathies, there are also a number of more complex tumor predisposition syndromes, such as neurofibromatosis I (von Recklinghausen disease), multiple endocrine neoplasias type 2A and 2B, familial medullary thyroid carcinoma, Stuge-Weber syndrome and neurocutaneous melanosis (Etchevers et al., 2006).

## Molecular basis of neurocristopathies

### Induction

The roles of Wnt, Fgf, Bmp, and Shh signaling families in neural crest induction/specification as well as their control by Hox transcription factor expression and retinoic acid gradients have been extensively studied and outlined (Duprez et al., 1999; Bastidas et al., 2004; Lewis et al., 2004; Cheung et al., 2005; Monsoro-Burq et al., 2005). Of particular importance to neural crest development is also the expression of several transcription factors of highly conserved families, such as Slug/Snail, Sox, Fox, and Pax in the neural folds or in tissues surrounding the sites of neural crest induction. In humans, mutations in *PAX3, MITF, SNAI2*, and *SOX10*, have all been directly linked to syndromic neurocristopathies (Dow et al., 1994; Pingault et al., 1998; Watanabe et al., 1998; Sanchez-Martin et al., 2002). *Slug/Snail* homologues are also expressed in the gastrulating mesoderm, and have been implicated in EMT (Locascio et al., 2002; Wakahashi et al., 2013).

### Migration

The migration of NCCs is a long and complex process, which starts with EMT, followed by delamination, and then migration along defined routes to various locations of the developing embryo. Experiments in mice have shown that non-canonical Wnt signaling via Wnt11 and its Frz7 receptor are required for the initiation and maintenance of the migratory behavior of NCC. Further, Sox9, Slug/Snail, Foxd3, and Sox10 also influence the cell-autonomous acquisition of a migratory behavior by NCC (Zhu et al., 2004; Cheung et al., 2005). The extracellular matrix, and the respective expression levels of fibronectins, vitronectins, laminins, and collagens further facilitate the migration of NCCs as they travel further from the neural tube and begin to enter various compartments (Britsch et al., 1998; Davy et al., 2004; Zhu et al., 2004; Pla et al., 2005).

### Differentiation

Naturally, the impressive variety of cell types generated by NCCs and the multitude of tissues to which these cell types contribute are both regulated by differentiation processes of great diversity and complexity. Importantly, the fate of NCCs is dependent on the intricate interactions between external stimuli and the internal environment of the cells. In addition, several factors have been shown to dedifferentiate NCCs into multipotent cells *in vitro*, which carries great significance for regenerative medicine and presents a line of research, which will be certainly expanded in coming years (Lee et al., 2007; Dupin and Coelho-Aguiar, 2013). A thorough review of all processes involved in NCC differentiation is beyond the scope of this article, but the three major signaling cascades are described in brief.

#### Signaling pathways of the pigmentation lineage

In the presence of endothelin-3 (Edn3), NCCs have been shown to differentiate into bipotent glia-melanocyte precursors and tripotent melanocyte-neural-adrenergic precursor cells (Stone et al., 1997; Dupin et al., 2000). Mutations of the endothelin B receptor (*Ednrb)* gene in mice has been shown to produce a recessive phenotype of spotting and megacolon, underlining the importance of endothelins in pigmentation and development of the gastrointestinal system (Hosoda et al., 1994). Mutations in human *EDNRB* have been linked to type 4 Waardenburg-Shah syndrome and Hirschsprung's disease, depending on the exact nature of the mutation (Puffenberger et al., 1994; Attie et al., 1995). EDNRB signaling is transduced to the interior of the cell via the Ras signaling pathway.

#### Signaling pathways of the neural lineages of the peripheral nervous system

In *Drosophila*, the *achaete-schute* complex, which encodes basic helix-loop-helix transcription factors, controls the development of the nervous system by regulating the differentiation of ectodermal cells into neuroblasts (Garcia-Bellido and de Celis, 2009). The homologs of these genes in mice are *Mash1 and Mash2* (Johnson et al., 1990; Parras et al., 2004). Mice with homozygous *Mash1* deletions die shortly after birth due to respiratory difficulties and the inability to suckle (Guillemot et al., 1995; Parras et al., 2004). These mice also lack parasympathetic and sympathetic ganglia, and the enteric nerves of the esophagus. HASH1, the human equivalent to Mash1, acts downstream of the PHOX2 transcription factors, which are expressed in all neurons in the noradrenergic synthesis pathway at some point (de Pontual et al., 2003; Pattyn et al., 2004).

#### Signaling pathways of the cardio-cephalic lineages

While it was long suspected that deficiencies in NCCs resulted in anomalies of the cardiac outflow tract and the caudal pharyngeal arches, it has recently been discovered that the pharyngeal endoderm is also required for survival and fate determination of cephalic NCCs (Couly et al., 2002). This appears to be due to the release of retinoic acid by the adjacent mesoderm and concomitant regulation of Hox gene activity, which determines rostrocaudal identity within surrounding cells and tissues (Halilagic et al., 2003; Bohnsack et al., 2004). Further, the production of developmental signaling molecules such as sonic hedgehog (Shh) and fibroblast growth factors (Fgfs) have also been implicated as controlling the processes of cardiac and cephalic NCC differentiation (Schneider et al., 2001; Abu-Issa et al., 2002).

## Ribosome biogenesis and the role of the ribosome in neurocristopathies

Interestingly, the recently improved molecular understanding of some neurocristopathies revealed a particular connection to ribosome biogenesis defects, which appear to particularly affect the survival of NCCs and is reviewed below. Ribosome biogenesis begins in the nucleus of eukaryotic cells when rRNA precursor molecules associate with both pre-ribosomal and ribosomal proteins (Venema and Tollervey, 1999). The entire process of ribosome biosynthesis involves all three RNA polymerases and the participation of well over 100 non-ribosomal proteins (Kressler et al., 2010). After synthesis, which requires transcription by RNA polymerase II, the ribosomal proteins are assembled in the cytoplasm and subsequently exported into the nucleus. As this is happening, the 45S pre-rRNA transcribed by RNA polymerase I undergoes a series of conformational changes and cleavages to form the 18, 5.8, and 28S rRNAs. In contrast, the 5S rRNA is independently transcribed by RNA polymerase III. The 18S rRNA and 32 ribosomal proteins combine to form the 40S small subunit, while the 5.8, 28, and 5S rRNAs combine with 47 ribosomal proteins to form the 60S large subunit. Both subunits are then transferred to the cytoplasm for final assembly (Johnson et al., 2002; Henras et al., 2008). Disruption of these processes results in nucleolar stress, which commonly precipitates G1 cell cycle arrest and cell death, via an increase in the activity of the tumor-suppressor Tp53.

**Figure 2:**
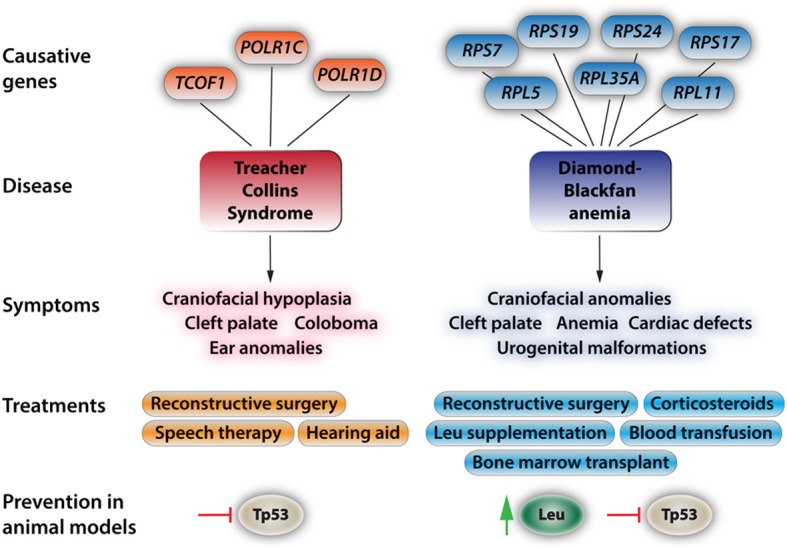
**Aspects of the two most prevalent and best-understood neurocristopathies/ribosomopathies, Treacher Collins Syndrome and Diamond-Blackfan anemia**. At the top, the diagram depicts known genes with causative mutations in the respective disorders. For more in depth reviews see Trainor (2010), Dauwerse et al. (2011), Payne et al. (2012), and Boultwood et al. (2013). The most-common symptoms are listed below. These symptoms may appear isolated or more commonly in conjunction with the other symptoms. Current treatments and approaches for ameliorating the effects of these syndromes. For the commonly found craniofacial dysplasias only reconstructive surgery is effectively used at this point. While leucine treatment has had positive results in human patients, amino acid treatments come with a bevy of side effects such as ketoacidosis, decreased blood sugar due to increased insulin release by the pancreas, complications of lung function, and hepatic encephalopathy. Recent studies in animal models have shown that Tp53 inhibition will at least partially rescue phenotypic deficiencies in TCS and that Tp53 inhibition and leucine treatment reduce the severity of the effects of DBA.

TCS syndrome and DBA are examples of well-characterized syndromes that display craniofacial hypoplasia and orofacial clefting with the underlying causes involving defects in ribosome generation (see Figure 2 for brief description). The *Tail-short* mice can be added to these examples as an animal model with midfacial clefting of partial penetrance (Morgan, 1950). TCS is a heterogeneous disorder, which was initially shown to arise from mutations in Treacle, a nuclear phosphoprotein, encoded by the *TCOF1* gene (Trainor et al., 2009). TCS causes dysplasia of the craniofacial structures including the bones of the face, the palate, external ear structures and the ear canals (Dauwerse et al., 2011). The disorder results in disrupted craniofacial development due to alterations in 28S ribosome biogenesis, and the resulting stabilization and cellular enrichment of Tp53 due to nucleolar stress. As a consequence, increased Tp53 levels lead to G1 cell cycle arrest and specific apoptosis of neuroepithelial cells, which results in hypoplastic neural crest-derived structures of the craniofacial skeleton (Jones et al., 2008; Sakai and Trainor, 2009). More recent work has identified cases of TCS in which human patients carry mutations in RNA polymerases I and III, providing further evidence that TCS is a neurocristopathic ribosomopathy (Dauwerse et al., 2011). Specifically, both heterozygous and homozygous carriers of mutant alleles in *POL1RC* and *POL1RD* were detected that displayed symptoms of TCS but did not express the mutant treacle protein (Dauwerse et al., 2011). Finally, it has been demonstrated that both genetic and pharmacological inactivation of Tp53 is capable of ameliorating the phenotypic defects associated with TCS via reduction of cyclin-G1 mediated cell cycle arrest and a reduction in Tp53 driven cell death of NCCs (Jones et al., 2008). Rescued mutants show improvements in craniofacial morphogenesis as well as an extended lifespan compared to their untreated counterparts. These improvements occur apart from the deficits shown in ribosome biogenesis, which suggests that Tp53 mediated cell cycle arrest is the primary pathological mechanism in mice affected with TCS (Jones et al., 2008).

DBA is a disease caused by mutations in any one of several ribosomal proteins, such as RPL15, RPL26, RPL35, RPL35a, RPLP2, RPS14, and RPS19, which lead to an imbalance between rRNA and ribosomal protein levels during construction of the ribosome (Uechi et al., 2008; Devlin et al., 2010; Horos and von Lindern, 2012; Landowski et al., 2013). Eventually, altered ribosome biosynthesis and the resultant accumulation of free ribosomal proteins leads to the stabilization and enrichment of Tp53, decreased proliferation, and increased apoptosis in a variety of tissues (Horos and von Lindern, 2012). DBA is an inherited disease with its primary symptoms being normochromic anemia, macrocytic anemia, diminished numbers of erythroid progenitor cells in the bone marrow, and reticulocytopenia, or decreased immature red blood cell count (Payne et al., 2012; Landowski et al., 2013). A subset of patients also display hypoplastic craniofacial structures, as well as malformations of the upper limb, heart and urogenital tract (Landowski et al., 2013). It has been demonstrated that haploinsufficiency of ribosomal proteins Rps14 and Rps19 causes symptoms resembling DBA in zebrafish such as anemia and craniofacial dysplasia, and also that treatment with L-Leucine reduces the severity of both anemia and developmental deficits (Payne et al., 2012; Boultwood et al., 2013). L-leucine induces protein synthesis primarily though induction of the mammalian target of rapamycin complex 1 (mTORC1) and the respective homologs in non-mammalian species. A considerable volume of research has uncovered in great detail the central role mTORC1 occupies in the regulation of cell growth and survival, however the mechanism by which mTORC1 is regulated by amino acids such as L-leucine is just beginning to be understood (Sengupta et al., 2010; Payne et al., 2012). The activation of mTORC1 leads to phosphorylation of two target proteins; eukaryotic translation initiation factor 4E-binding protein 1 (Eif4Ebp1), which is critical for cap-dependent mRNA translation, and S6 kinase1 (S6K1), which promotes mRNA translation via regulation of the proteins involved (Ruvinsky and Meyuhas, 2006; Khanna-Gupta, 2011). It appears that in Rps14- and Rps19-deficient zebrafish the Torc1 pathway becomes activated upon nucleolar stress response (Payne et al., 2012). L-leucine treatment in Rps14- and Rps19-deficient zebrafish causes an increase in S6K1 phosphorylation compared to untreated animals, which already have higher than normal levels of S6K1 activity, meaning that over-activation of the Torc1 pathway is likely necessary in order to overcome the deficiencies in these animals (Payne et al., 2012). In human hematopoietic CD34^+^ stem cells, deletion of RPS14 and RPS19 cause Tp53 accumulation specifically in erythroid progenitor cells, which results in cell cycle arrest and apoptosis in specific cell types and this effect can be reversed by inhibition of Tp53 activity (Dutt et al., 2011). Perhaps most interestingly, increases in Tp53 activity have been associated with significant decrease in S6K1 phosphorylation as well as an increase in deactivation of Eif4Ebp1 via dephosphorylation (Horton et al., 2002; Dutt et al., 2011). However, mRNA levels of Tp53 target genes remains unchanged during L-leucine treatment, suggesting that the amino acid treatment acts outside of the Tp53 pathway (Payne et al., 2012). Additionally, human DBA patients of the same family carrying the same mutation can present themselves with drastically different phenotypic effects, further illustrating the complexity of the disease. While DBA patients and animal models for DBA display deficits in neural crest-derived structures, the molecular studies on its neurocristopathic aspects have not yet been performed. Consequently, DBA may be classified as a ribosomopathy with neurocristopathic aspects.

Finally, recent research has confirmed that *Tail-short* (*Ts*^+/−^) mice, which display a partially penetrant median facial cleft, carry dominant mutations in the ribosomal protein Rpl38 (Kondrashov et al., 2011). Moreover, this landmark study has clearly shown that Rpl38 can mediate the selective translation of *Hox* mRNA transcripts, intriguingly, providing the first evidence that ribosomal proteins exert a regulatory function in the tissue-specific translation of certain mRNAs (Kondrashov et al., 2011). Specifically, the detailed quantification of mRNA transcripts of *Hox* genes revealed no changes in overall expression levels in the *Ts* mutants, but certain *Hox* transcripts show significant changes in protein levels in mutant animals. The study strongly suggests that certain *Hox* genes require normal Rpl38 functionality for proper translation. Also, a genetic screen displays remarkable dynamic regulation of the ribosomal proteins throughout the vertebrate embryo during development, perhaps suggesting that tissue specific phenotypes, such as the ribosomopathies described here, are the result of alterations in specific ribosomal associated proteins required for translation of specific morphogenetic mRNAs (Kondrashov et al., 2011).

## Cell cycle regulation by the TP53-MDM2 loop

The processes of cell growth and proliferation are intrinsically linked and both are highly dependent on proper protein synthesis mediated by a functional ribosomal machinery. In tissues, organs, and entire organisms, the maintenance of an appropriate cell number set by the proper balance of apoptosis and proliferation is critical for correct function and survival. For instance, the rate of proliferation is regulated at the level of cell cycle progression, which is tightly controlled at each phase, most notably at the G1/S transition point. In response to a variety of extracellular and intracellular signals, the cell can divide, become quiescent, or even differentiate (Norbury and Nurse, 1992; Du and Stillman, 2002). Many types of tumorigenic cancers arise from loss of control at this checkpoint, often resultant of improper regulation of proto-oncogenes or tumor-suppressor genes (Green and Evan, 2002; Vousden, 2002). For instance, the tumor-suppressor protein TP53 plays a central role in the G1 to S transition. Characteristically, mutations in *TP53* are found in more than 50% of all human cancers, and modulators of TP53 have been implicated in many other cancers. Notably, the *MDM2* gene, which encodes a TP53 inhibitor, is altered in nearly 10% of all non-TP53 originating cancer types (Kubbutat et al., 1997; Momand et al., 1998; Toledo and Wahl, 2006). In the cell, TP53 levels are usually kept low, due to continuous degradation by the E3-ligase MDM2, which tags TP53 for proteolysis by the proteasome (Haupt et al., 1997; Honda et al., 1997). Further, MDM2 is transcriptionally activated by TP53, creating a self-regulating negative feedback loop (Michael and Oren, 2003). During nucleolar stress, which may be triggered by a disruption in ribosome biogenesis, MDM2 becomes inactivated by its association with ribosomal proteins L5 and L11, and loses its ability to ubiquitinate TP53 (Lohrum et al., 2003; Zhang et al., 2003; Dai and Lu, 2004). Consequently, TP53 levels increase within the cell leading to G1 cell cycle arrest, apoptosis, or senescence. (Vogelstein et al., 2000).

## Is there a specific sensitivity of the neural crest to aberrant cell cycle regulation and altered ribosome biogenesis?

As outlined earlier, during EMT, migration, and differentiation of NCCs, several signaling pathways integrate to properly regulate cellular behavior and fate determination. Defective ribosome biosynthesis will not only impair cell survival due to Tp53 mediated cell-cycle arrest and apoptosis, surviving cells will be unable to produce new ribosomes as effectively thus limiting their capacity for creating new proteins necessary for such tightly orchestrated developmental events. It is conceivable that the specific program of EMT and long-range migration inherent to NCCs, imposes a particular burden on these cells to command a well-functioning biosynthetic machinery and thus a particular sensitivity to defects in ribosome biosynthesis. Intriguingly, studies in the slime mold *Dictyostelium*, have demonstrated the requirement for changes to the ribosomal protein profiles to transition cells from the multicellular fruiting body to unicellular vegetative cells, providing a possible analog for developmental changes that also govern NCCs (Ramagopal, 1992). Furthermore, ribosomal expression screens have revealed a dynamic regulation of the individual ribosomal proteins within the developing vertebrate embryo, suggesting that heterogeneous ribosomal protein populations are necessary for proper development of different tissues and structures in vertebrates (Kondrashov et al., 2011). To further explore these interesting questions, future studies may focus on isolating NCCs of different states, such as premigratory and migratory populations, using cell-sorting approaches and compare the composition of preribosomal and ribosomal proteins found in these cells. The fact that NCCs undergo rapid developmental transitions and precise levels of required proteins are needed for each step of the process, suggests that alterations in the ability to rapidly manufacture for instance signaling molecules or cytoskeletal components may have large-scale consequences.

## The *manta-ray* mutant: a novel model for the study of ribosomopathies and neurocristopathies in mice

The intricate and complex interactions between ribosome biogenesis defects and neurocristopathies can be best elucidated through the generation and analysis of suitable animal models. Consequently, the search for such models has to be an important component of future research efforts. Recently, we identified in a forward genetic screen in mice a line with pronounced craniofacial deficits inherited in an autosomal recessive pattern (Zarbalis et al., 2004). The line was named *manta-ray (mray)* due to its unique craniofacial morphology characterized by a median cleft of the maxillae and secondary palate. The palatal shelves of affected mutants elevate, but do not display proper outgrowth or fusion at the midline at E14.5. The nasal septum and other facial midline structures are reduced in size, as are the forebrain, hindbrain, and the majority of other tissues and organs of homozygous *mray* mutants. Positional cloning efforts revealed the causative mutation in the gene encoding the WD-40 domain-containing factor p21-activated kinase 1 inhibitory protein 1 (Pak1ip1) (Ross et al., 2013). Originally, the Pak1ip1 homologue Skb15 was identified in fission yeast (*Schizosaccharomyces pombe*) and characterized as a negative regulator of the p21-activated kinase, Shk1 (Kim et al., 2001). P21 activated kinases have been implicated in a myriad of cellular functions but are most often associated with their roles in cell polarity and as organizers of cytoskeletal assembly (Daniels and Bokoch, 1999; Ong et al., 2011). Skb15 was shown to be essential for cytoskeletal regulation as well as coordinating the process of cytokinesis (Kim et al., 2001). Perhaps most interestingly, it was shown that mouse Pak1ip1 was able to serve as a functional substitute for Skb15 in yeast cells, indicating that Pak1ip1 function remained highly conserved in species separated by approximately 1.5 billion years of evolution (Kim et al., 2001). Several years passed without any significant advancement of knowledge on the function of Pak1ip1 and its homologues, until a 2007 study characterized Skb15 and its homologue in budding yeast (*Saccharomyces cerevisiae*), Mak11, as 60S ribosomal assembly factors (Saveanu et al., 2007). Mak11 was found in association with the pre-60S assembly factor, Rlp24, an interaction that was strengthened when another pre-60S factor, Nog11, was depleted (Saveanu et al., 2003, 2007). Most importantly, the loss of Mak11 interfered with the maturation of 60S rRNA, resulting in G1 cell cycle delay and an increase in cell death. In light of these findings, the interaction of Pak1ip1 with Pak1 and the cellular events it mediates through this function appear secondary to its role in ribosome assembly considering the paramount cellular role of this major metabolic activity. However, it was not until 2011 that PAK1IP1 was confirmed as a nucleolar protein in human cells, required for rRNA processing, and acting as a nucleolar stress response signal with the capacity to activate the TP53-MDM2 loop and downstream events (Yu et al., 2011). Specifically, it was shown that both PAK1IP1 overexpression and siRNA knockdown cause an increase in MDM2 inhibition, and thus TP53 activation, leading to G1 cell cycle arrest and the cessation of cell proliferation.

**Figure 3:**
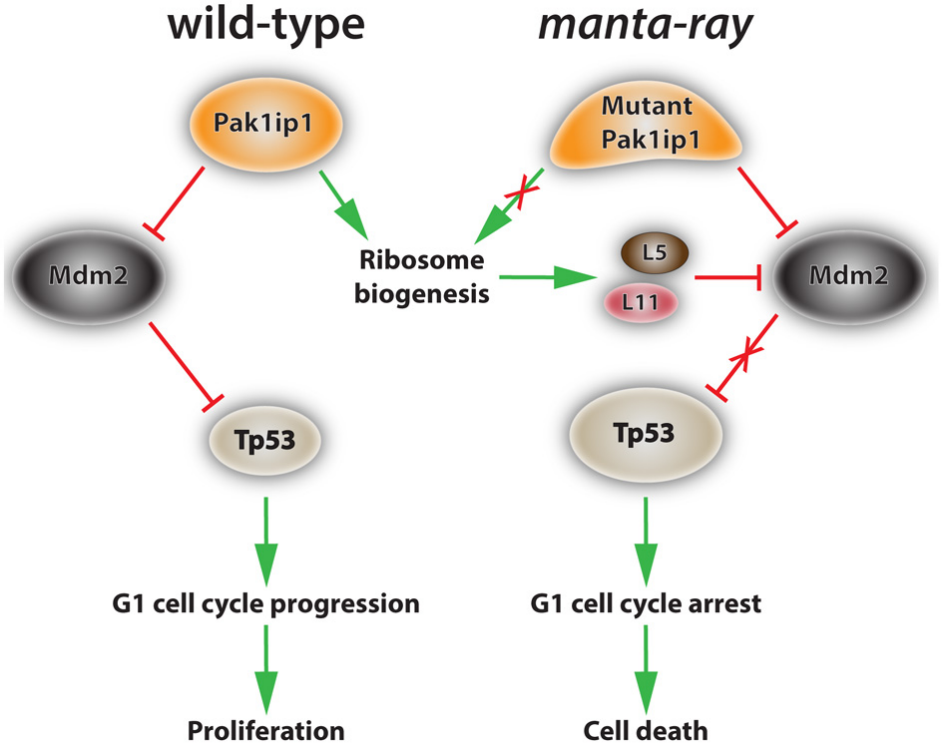
**Proposed mechanism of action of Pak1ip1 in both wild-type and homozygous *manta-ray* mutants**. In the wild-type (left) Pak1ip1 acts as an inhibitor of the E3 ubiquitin ligase Mdm2, which acts as an inhibitor of Tp53 activity through an auto-regulatory feedback loop. Mdm2 targets Tp53 for degradation by the proteasome, blocks its transcriptional activity, and facilitates its export from the nucleus. Low levels of Tp53 activity promote cell cycle progression at the G1 checkpoint and cellular proliferation. In the *manta-ray* mutant (right) Pak1ip1 causes altered 60S ribosome biogenesis, resulting in nucleolar stress and the accumulation of freely circulating ribosomal proteins L5 and L11, which inhibit Mdm2 activity. This series of events leads to an increase in Tp53 levels, G1 cell cycle arrest, and subsequent cell death, which appears to particularly affect aspects of the cranial neural crest.

Our recent study in *mray* mice, so far the only animal model available with loss of Pak1ip1 function, provided further insight into the developmental role of this molecule (Ross et al., 2013). Most importantly, homozygous *mray* mutants show an upregulation of Tp53, a hallmark of ribosomopathies, while the Tp53-Mdm2 interaction does not appear to be disrupted, as revealed by co-immunoprecipitation experiments. Apparently Pak1ip1 loss-of-function can adversely affect ribosome biogenesis and trigger a Tp53 response without acting directly through Mdm2, but presumably through increases in the levels of freely circulating Rpl5 and Rpl11, which function as Mdm2 inhibitors (Figure 3).

The analysis of *mray* mice further added to our understanding of the connections between ribosomopathies and neurocristopathies with important conclusions to be drawn. First, despite ribosome biogenesis being a ubiquitous process required by every cell, the phenotypic outcomes by mutating individual components of this biosynthetic machinery, which involves the coordinated function of at least 200 proteins, can be vastly different. Second, neural crest-derived structures are particularly affected by altered ribosome biogenesis, possibly due to a specific sensitivity imposed by their developmental origin. Third, neural crest-derived structures will present with different phenotypic outcomes depending on which individual components of ribosome biosynthesis are impaired. While the causes of this variation are not understood, it is conceivable that individual subpopulations of NCCs depend to a different degree or at different developmental stages on ribosome biosynthesis, specific preribosomal and ribosomal factors are required to a different extent by certain NCC populations, or any combination thereof. The generation and analysis of additional animal models for ribosomopathies will certainly shed more light to these interesting questions.

## Future directions and proposed experiments

Advancements in the understanding of how ribosomopathies affect craniofacial development will likely come from two major avenues of research. First, it will be necessary to determine exactly how the composition of ribosomal proteins and associated ribosomal cofactors change in NCCs at different developmental stages. These studies will incorporate advanced proteomic techniques capable of detecting changes in the proteome of NCCs in order to reveal dynamic changes in ribosomal protein regulation during neural crest development. Second, the study of suitable animal models with ribosomal disorders and associated craniofacial dysplasias will be a necessary component for progress in the field. Tissue specific gene ablation or overexpression, directed mutagenesis, or perhaps crossing of existing animal models would provide greater insight as to how various perturbations in ribosomal function and regulation affect development of the craniofacial structures.

In order to fully examine the role Pak1ip1 plays in cellular function and particularly in the function of NCCs, several experiments are necessary using the *manta-ray* mice. The establishment of polyribosomal profiles for mutant mice and their comparison to controls will further illuminate the specific biosynthetic steps impaired in the mutant. The use of either pharmacological or genetic inhibition/downregulation of Tp53 in affected mray mutants and the analysis of the phenotypic consequences, will inform us as to whether the phenotypic alterations in the mutant are exclusively a consequence of Tp53 activation. Furthermore, in addition to measuring ribosome biosynthesis directly, it will be pertinent to measure S6K activity as a measure of translation, as well as to investigate the effects of amino acid treatments, such as L-leucine on the *manta-ray* phenotype.

### Conflict of interest statement

The authors declare that the research was conducted in the absence of any commercial or financial relationships that could be construed as a potential conflict of interest.
